# Supplementary material for: Identification of Clinical and Socioeconomic Predictors of Adjuvant Therapy after Trans-Oral Robotic Surgery in Patients with Oropharyngeal Squamous Cell Carcinoma
Source: Cancers (Basel). 2020 Sep 1;12(9):2474. doi: 10.3390/cancers12092474 (PMC7565070; doi:10.3390/cancers12092474)
Supplement: Supplementary file 1 [file cancers-12-02474-s001.pdf]

cancers

Article

Identification of Clinical and Socioeconomic Predictors of Adjuvant Therapy After Trans-Oral Robotic Surgery in Patients with Oropharyngeal SCC

Sujith Baliga, Brett Klamer, Sachin Jhawar, Mauricio Gamez, Darrion Mitchell, Adriana Blakaj, John Grecula, Ulysses Gardner, Khlaed Dibs, Matthew Old, Nolan Seim, Stephen Kang, Ricardo Carrau, Amit Agrawal, Vidhya Karivedu, Priyanka Bhateja, Enver Ozer , James Rocco, Marcelo, Bonomi and Dukagjin Blakaj

SupplementaryMaterials:

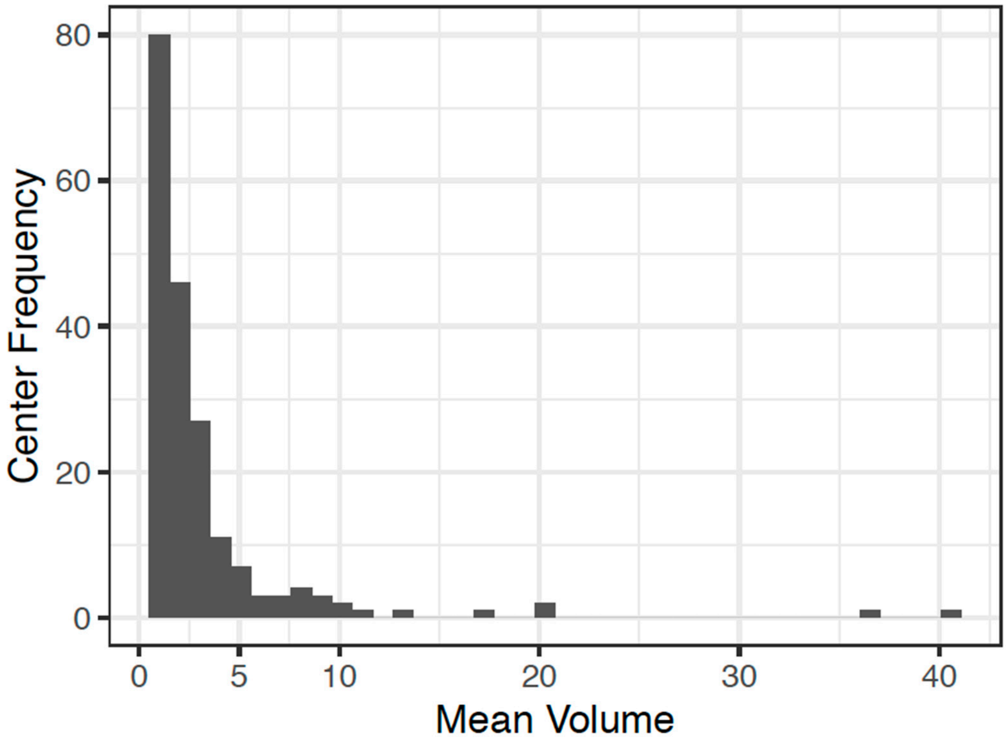

Figure S1. Relationship between mean center volume of TORS and center frequency.

Predictors	Estimate (95% CI)
Sociodemographic:	
• Age	-0.03 (-0.1, 0.04)
Sex	
• Male	Reference
• Female	0.1 (-1.49, 1.69)
Race	
• White	Reference
• Black	-0.25 (-2.74, 2.24)
• Hispanic	3.85 (0.42, 7.29)
• Other/Unknown	2.43 (-0.97, 5.83)
Insurance status	
• Private insurance	Reference
• Medicare	1.56 (0.06, 3.06)
• Medicaid	-0.1 (-2.54, 2.34)
• Other/Unknown	-0.39 (-3.02, 2.25)
Clinical:	
Chemotherapy	
• No	Reference
• Yes	4.08 (2.75, 5.41)
ECE	
• RLN+, ECE-	Reference
• RLN+, ECE+	1.46 (0.19, 2.73)
• RLN+, ECE unknown	3.11 (0.25, 5.97)
• RLN-	2.12 (-0.44, 4.68)
LVSI	
• Negative	Reference
• Unknown	1.02 (-0.62, 2.65)
• Positive	-0.17 (-1.32, 0.98)
N Stage	
• 0	Reference
• 1	-1.79 (-3.94, 0.35)
• 2	-0.32 (-2.85, 2.2)
• 2A	-1.24 (-3.6, 1.11)
• 2B	-0.75 (-2.81, 1.3)
• 2C/3	0.28 (-2.72, 3.29)
Margin	
• Negative	Reference
• Positive	2.62 (1.12, 4.11)

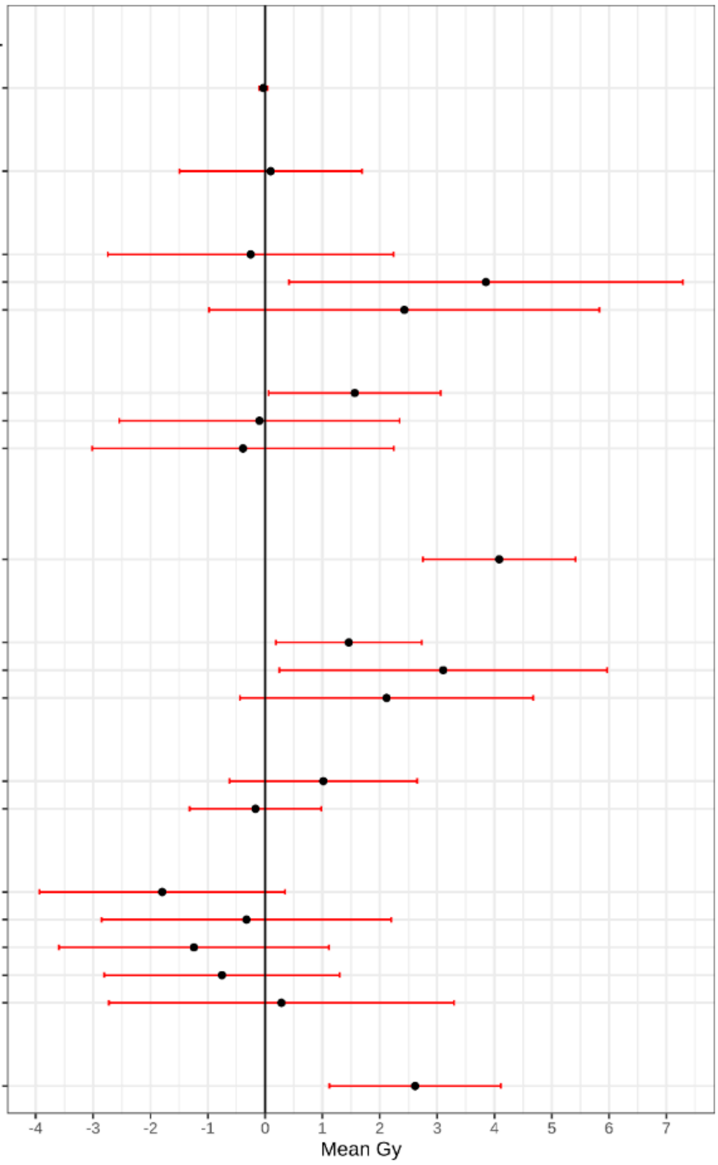

Figure S2. Forrest plot depicting mean change of estimated radiation dose among patients receiving doses of 50–70 Gy.
